# Distribution of manganese and other biometals in *flatiron* mice

**DOI:** 10.1007/s10534-015-9904-2

**Published:** 2015-12-22

**Authors:** Young Ah Seo, Jamal A. Elkhader, Marianne Wessling-Resnick

**Affiliations:** Department of Genetics and Complex Diseases, Harvard T.H. Chan School of Public Health, 665 Huntington Avenue, Boston, MA 02115 USA

**Keywords:** *Flatiron* mice, Ferroportin, Slc40a1, Manganese, Iron, Copper, Zinc

## Abstract

*Flatiron* (*ffe*) mice display features of “ferroportin disease” or Type IV hereditary hemochromatosis. While it is known that both Fe and Mn metabolism are impaired in *flatiron* mice, the effects of ferroportin (Fpn) deficiency on physiological distribution of these and other biometals is unknown. We hypothesized that Fe, Mn, Zn and/or Cu distribution would be altered in *ffe/*+ compared to wild-type (+*/*+*)* mice. ICP-MS analysis showed that Mn, Zn and Cu levels were significantly reduced in femurs from *ffe/*+ mice. Bone deposits reflect metal accumulation, therefore these data indicate that Mn, Zn and Cu metabolism are affected by Fpn deficiency. The observations that muscle Cu, lung Mn, and kidney Cu and Zn levels were reduced in *ffe/*+ mice support the idea that metal metabolism is impaired. While all four biometals appeared to accumulate in brains of *flatiron* mice, significant gender effects were observed for Mn and Zn levels in male *ffe/*+ mice. Metals were higher in olfactory bulbs of *ffe/*+ mice regardless of gender. To further study brain metal distribution, ^54^MnCl_2_ was administered by intravenous injection and total brain ^54^Mn was measured over time. At 72 h, ^54^Mn was significantly greater in brains of *ffe/*+ mice compared to +*/*+ mice while blood ^54^Mn was cleared to the same levels by 24 h. Taken together, these results indicate that Fpn deficiency decreases Mn trafficking out of the brain, alters body Fe, Mn, Zn and Cu levels, and promotes metal accumulation in olfactory bulbs.

**Electronic supplementary material:**

The online version of this article (doi:10.1007/s10534-015-9904-2) contains supplementary material, which is available to authorized users.

## Introduction

Ferroportin (Fpn; SLC40A1) is a metal exporter involved in the assimilation of dietary Fe and Mn (Seo and Wessling-Resnick 2015). Several lines of evidence suggest it also may function in the transport of additional metals. Cu treatment induced Fpn expression and was associated with Fe efflux in J774 macrophage cells (Chung et al. 2004). Troadec et al. (2010) observed that Zn and Cd also induced Fpn expression and that this effect was associated with the binding of Metal Transcription Factor-1 (MTF1) to the Fpn promoter. Moreover, Fpn appeared to protect cells from Zn toxicity in this study (Troadec et al. 2010). Others have shown that Fpn expression in *Xenopus* oocytes stimulated efflux of ^55^Fe, ^65^Zn, and ^57^Co and that hepcidin, a hormone peptide that down-regulates Fpn (Nemeth et al. 2004), blocked export of these metals (Mitchell et al. 2014). These independent lines of evidence lead to the hypothesis that Fpn plays a role in the metabolism of metals in addition to Fe and Mn.

Patients with mutations in the Fpn gene develop hereditary hemochromatosis (HH) type 4, also called “ferroportin disease”, which is associated with Fe-loading and restricted erythropoiesis (Pietrangelo 2004). Substantial effort has been directed towards developing in vivo models of ferroportin disease. Mutation of the zebrafish homolog causes defective Fe transport from the yolk sac to embryo so it is difficult to translate information about the human disease (Donovan et al. 2000). *Fpn* null mice display embryonic lethality, and heterozygous *Fpn*^*null/*+^ mice do not load Fe although they do have mildly impaired Fe homeostasis (Donovan et al. 2005). Disruption of intestinal *Fpn* confirmed its role in Fe absorption, but these mice were severely anemic (Donovan et al. 2005). Hepatocyte-specific *Fpn* knockout mice retained liver Fe, but did not fully recapitulate the phenotype of human ferroportin disease due to compensating enhancement of intestinal absorption (Donovan et al. 2005). Tissue specific disruption of *Fpn* in macrophages also has been established, but these mice also developed severe anemia with Fe retention due to impaired mobilization after recycling of Fe (Zhang et al. 2012). *Flatiron* (*ffe*) mice have provided a more useful genetic model that fully recapitulates ferroportin disease. The *flatiron* mutation (H32R) was identified in a screen for ethylnitrosourea-induced mutations that affected embryonic formation (Zohn et al. 2007). Although embryos homozygous for this mutation showed severe anemia and mid-gestational lethality, heterozygous animals develop the phenotypic characteristics of ferroportin disease, with reduced hematocrit, hepatic Fe-loading, high serum ferritin, and low transferrin saturation (Zohn et al. 2007). Furthermore, the *flatiron* mutation H32R shows dominant negative effects much like other missense mutations in Fpn causing the human ferroportin disease (Zohn et al. 2007). More recent studies from our laboratory have shown that Fpn deficiency impaired Mn metabolism in *flatiron* mice, a genetic model of Fpn deficiency (Seo and Wessling-Resnick 2015). Fpn deficiency reduced intestinal Mn absorption, and lowered blood, liver, and bile Mn levels (Seo and Wessling-Resnick 2015). Like Fe, Mn is both required yet toxic when present in excessive amounts. In particular, accumulation of Mn in the brain produces neurotoxic effects disrupting motor function and behavior (Guilarte 2010).

While the role of Fpn in Fe metabolism has been established, and its influence on Mn homeostasis begins to be appreciated, relatively little is known about the potential impact of Fpn on the distribution of other metals or how ferroportin disease might alter metal metabolism. Therefore, we undertook this study to determine the distribution of Mn, Fe, Zn, and Cu in relevant tissues and organs collected from heterozygous *ffe/*+ mice. In addition, we evaluated accumulation of brain Mn after intravenous administration of ^54^Mn in *flatiron* and wild-type mice.

## Materials and methods

### Animal care and procedures

This study was performed in strict accordance with the recommendations in the Guide for the Care and Use of Laboratory Animals of the National Institutes of Health. The protocol used for these studies (Animal Experimentation Protocol IS00000040) was approved by the Harvard Medical Area Animal Care and Use Committee. *Flatiron* (*ffe/*+) mice were kindly provided by Dr. Irene E. Zohn (University of Colorado at Denver and Health Sciences Center, CO). All mice used for these studies were on the 129S6/SvEvTac background. The +*/*+ and *ffe/*+ groups were verified by PCR genotyping (Zohn et al. 2007). Weanling mice were fed a diet containing 50 mg Fe/kg, 35 mg Mn/kg, 56 mg Zn/kg, and 10.5 Cu mg/kg (TD120518, Harlan Teklad) until 15 weeks of age. Levels of metals in the diet were as recommended by American Institute of Nutrition (Reeves et al. 1993). For trace element analysis, 10 mice/group (6 male and 4 female) were used. Mice were euthanized under isoflurane inhalation (5 %) followed by cardiac puncture and exsanguination prior to tissue collection. All experiments were carried out between 12–3 p.m. in order to avoid circadian effects on Fe metabolism. Hematocrit and tissue nonheme Fe concentrations were measured as previously described (Seo and Wessling-Resnick 2015).

Mn brain levels were determined after intravenous administration of ^54^Mn at 15 weeks of age. ^54^MnCl_2_ (Perkin Elmer/NEN, Boston) was diluted to 200 µCi/mL in phosphate-buffered saline (PBS), and 1.5 mL/kg was injected into the tail vein under anesthesia with isoflurane. For brain ^54^Mn levels after intravenous studies, 5 mice/group (3 male and 2 female) were used. Mice were euthanized by isoflurane overdose 1, 24, or 72 h post-dose of ^54^MnCl_2_ to collect blood and brain tissues. Radioactivity was quantified in a Packard gamma counter (Cobra Quantum, Packard Instrument, Downers Grove, IL).

### Trace element analysis

All samples were handled with special care in order to avoid environmental contamination. Blood was collected in an anticoagulant (EDTA) tube using heart puncture with a sterile syringe. After centrifugation at 1000×*g* for 10 min at 4 °C, the top yellow plasma layer was collected, white buffy layer (leukocytes) was discarded, and erythrocytes were collected. Bone samples were taken from femurs and excluded the marrow. Muscle samples were taken from quadriceps.

Samples were analysed for metals by inductively coupled plasma mass spectrometry (ICP-MS) (Trace Metals Laboratory, Harvard School of Public Health, Boston, MA) as described previously (Seo and Wessling-Resnick 2015). The internal standard was 50 ppb Indium. Briefly, tissue samples taken from mice were digested with 2 mL/g total wet weight nitric acid (BDH ARISTAR^®^ ULTRA) for 24 h, and then digested with 1 mL/g total wet weight hydrogen peroxide (BDH Aristar^®^ ULTRA) for 24 h at room temperature. Specimens were preserved at 4 °C until quantification of metals. Ultrapure water was used for final sample dilution.

### Statistical analysis

Data shown are the mean ± SEM. Statistical comparisons were determined with Student’s t test and two-way ANOVA followed by Bonferroni post hoc test as appropriate (Prism Graph Pad, Berkeley, CA). Differences were considered significant at P < 0.05.

## Results

### Physiological and haematological characteristics of flatiron mice

 For this study, heterozygous *flatiron* and wild-type (+*/*+) siblings were fed a diet containing 50 mg Fe/kg, 35 mg Mn/kg, 56 mg Zn/kg, and 10.5 Cu mg/kg (TD 120518, Harlan Teklad). At 15 weeks of age, mice were humanely sacrificed to determine the distribution of metals. Physiological and hematological characteristics of *ffe/*+ mice were compared to +*/*+ littermates. Body and organ weights in *ffe/*+ mice were similar to +*/*+ mice (Table 1) although *ffe/*+ brain weighed slightly less compared to +*/*+ mice. As expected, *ffe/*+ mice had reduced hematocrit values (*P* < 0.05) and higher non-heme Fe levels in both liver and spleen (*P* < 0.05) compared to +*/*+ mice (Table 2). There was no difference in food consumption between the two groups (data not shown). Consistent with previous studies (Seo and Wessling-Resnick 2015; Zohn et al. 2007), these data support the *flatiron* mouse phenotype associated with ferroportin disease (Pietrangelo 2004). No significant difference was detected Cu or Zn levels in erythrocytes versus plasma between the two groups (data not shown). Adjusting for lower haematocrit, the level of circulating Mn associated with the red blood cell fraction was significantly reduced in *ffe/*+ mice compared to +*/*+ mice (2.5 µg/L vs. 2.9 µg/L, *P* = 0.011). This is consistent with our previous studies showing that *flatiron* mice displayed reduced total blood Mn levels at 6, 8, and 16 weeks of age (Seo and Wessling-Resnick 2015).

**Table 1:** Physiological characteristics of *flatiron (ffe)* mice

	+/+	n	*ffe*/+	n
Body weight (g)	25.8 ± 1.806	10	25.5 ± 1.470	10
Brain weight (g)	0.45 ± 0.005	10	0.44* ± 0.003	10
Bone weight (g)	0.14 ± 0.008	10	0.12 ± 0.004	10
Pancreas weight (g)	0.10 ± 0.008	10	0.09 ± 0.009	10
Liver weight (g)	0.88 ± 0.054	10	0.84 ± 0.100	10
Heart weight (g)	0.13 ± 0.008	10	0.13 ± 0.010	10
Lung weight (g)	0.13 ± 0.004	10	0.13 ± 0.004	10
Kidney weight (g)	0.28 ± 0.040	10	0.29 ± 0.025	10
Spleen weight (g)	0.04 ± 0.003	10	0.04 ± 0.002	10

Data are presented as the mean ± SEM; n, no. of mice

* P < 0.05 between control (+/+) and flatiron mice (ffe/+)

**Table 2:** Haematological characteristics of *flatiron (ffe)* mice

	+*/*+	n	*ffe/*+	n
Hematocrit (%)	49.8 ± 0.416	10	47.2* ± 0.800	10
Liver nonheme iron (μg/mL)	112.9 ± 10.10	10	151.9* ± 12.19	10
Spleen nonheme iron (μg/mL)	571.6 ± 3.149	10	587.0* ± 1.066	10

Data are presented as the mean ± SEM; n, no. of mice

* P < 0.05 between control (+/+) and flatiron mice (ffe/+)

### Metal distribution in bone and muscle of flatiron mice

**Fig. 1:**
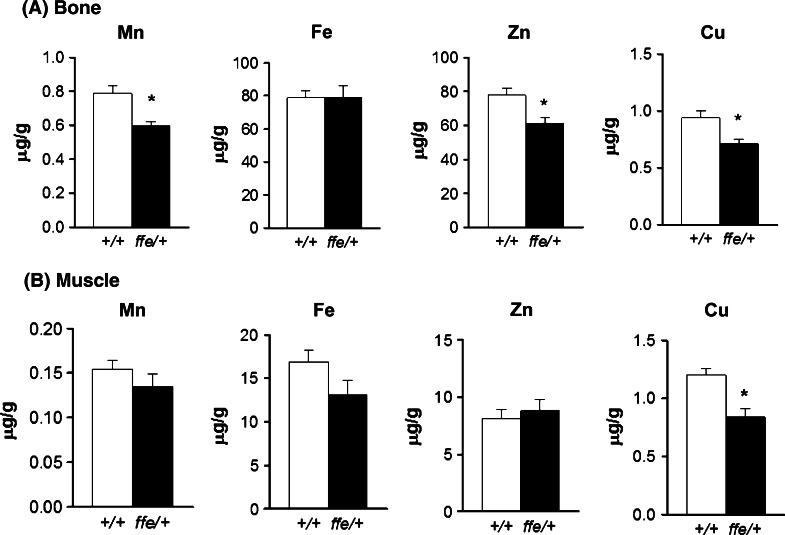
Metal levels in bone and muscle of wild-type and *flatiron* mice. Metal levels were measured by ICP-MS in femurs (**a**) and quadriceps muscle (**b**). *Empty and closed bars* represent +*/*+ and *ffe/*+ mice, respectively. Data are mean ± SEM (n = 6 male and 4 female mice for each genotype). *P < 0.05 between +*/*+ versus *ffe/*+ mice; *t* test

Bone has been used to assess metal accumulation in humans and mice because metals are incorporated in calcified tissues and therefore subjected to bone turnover (Gdula-Argasinska et al. 2004). Thus, we measured the levels of metals in femur and compared these values to quadriceps muscle. Bone Mn (0.596 vs. 0.788 mg/kg; *P* = 0.002), Zn (61.079 vs. 77.840 mg/kg; *P* = 0.008), and Cu (0.716 vs. 0.941 mg/kg; *P* = 0.008) levels were significantly reduced in *ffe/*+ mice compared to +*/*+ mice (Fig. 1a). Amongst the metals studied, only Cu levels were significantly reduced in *ffe/*+ muscle relative to wild-type (Fig. 1b).

### Tissue metal levels in flatiron mice

**Fig. 2:**
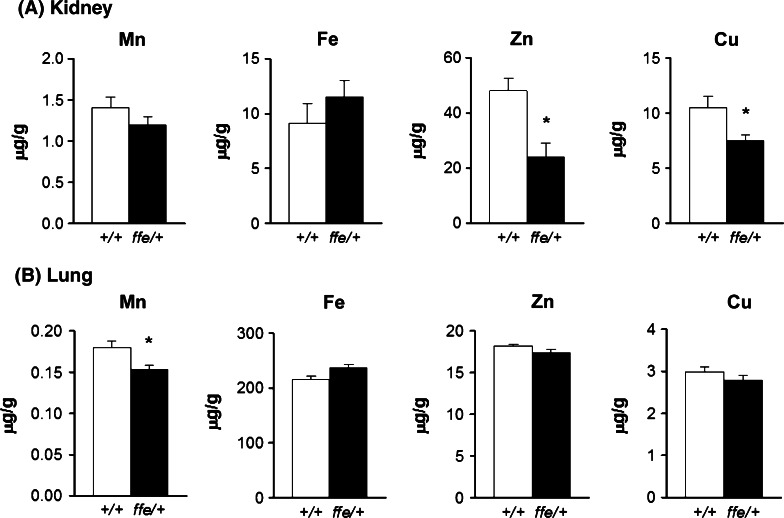
Kidney and lung metal levels in *ffe/*+ mice. ICP-MS was used to determine levels of Mn, Fe, Zn and Cu kidney (**a**) and lung (**b**). *Empty and closed bars* represent +*/*+ and *ffe/*+ mice, respectively. Data are mean ± SEM (n = 8–10 mice/group). *P < 0.05 between +*/*+ versus *ffe/*+ mice; *t*-test

To further explore metal distribution, levels of Mn, Fe, Zn and Cu were measured in multiple organs. No differences in Mn, Zn, or Cu were detected in spleen, heart, and pancreas (data not shown). However, kidney Zn (24.0 vs. 48.0 mg/kg; P = 0.002) and Cu (7.5 vs. 10.5 mg/kg; P = 0.018) were reduced in *ffe*/+ mice compared to +*/*+ mice (Fig. 2a). Of note, lung Mn levels were significantly reduced in *ffe/*+ mice relative to +*/*+ mice (0.153 mg/kg vs. 0.180 mg/kg; *P* = 0.01) (Fig. 2b). It is important to note that the metal content in multiple organs were similar between +*/*+ siblings and values reported in previous studies of mice (summarized in Supplemental Table 1).

### Metal distribution in brain of flatiron mice

**Fig. 3:**
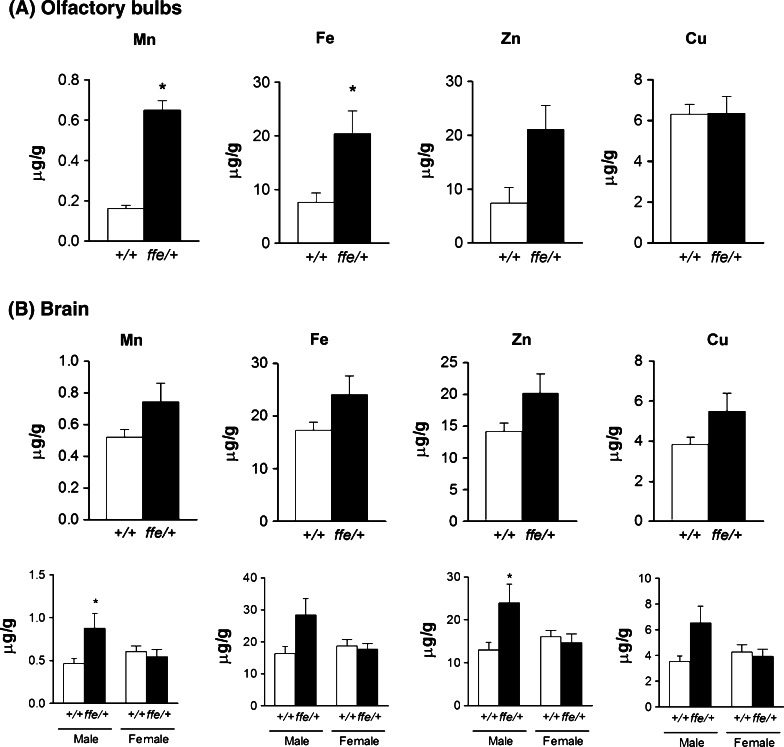
Metal levels in brain of wild-type and *flatiron* mice. Olfactory bulbs (**a**) from 3 to 4 mice of wild-type and *flatiron* mice were pooled and determined for metal levels by ICP-MS. The rest of the brain (**b**) was determined for metal levels by ICP-MS. *Empty and closed bars* represent +*/*+ and *ffe/*+ mice, respectively. Data are mean ± SEM (n = 10 mice/group in panel **a**; 6 male and 4 female per genotype in panel** b**). *P < 0.05 between +*/*+ versus *ffe/*+ mice; *t*-test

Levels of each of the metals were determined for brain. In addition, olfactory bulbs were dissected and measured separately from the rest of the brain since the olfactory bulb is known to be an important brain region for uptake and/or accumulation of metals (Sunderman 2001). Indeed, *ffe/*+ olfactory bulbs had increased content of Mn (0.6496 vs. 0.1616 mg/kg; *P* = 0.0006) and Fe (20.4 vs. 7.58 mg/kg; *P* = 0.05) compared to +*/*+ olfactory bulbs (Fig. 3a). Although not statistically different, Zn levels in olfactory bulbs were ~2.8 fold higher in *ffe/*+ mice compared to +*/*+ mice (21.08 vs. 7.41 mg/kg; *P* = 0.06). Generally, the brain metal content in *flatiron* mice was higher for all of the tested metals, with significant gender differences observed for Mn and Zn levels, which were greater in male *ffe/*+ mice compared to male +*/*+ mice (Fig. 3b). There were no significant gender differences between male and female wild-type mice, or between *ffe/*+ and +/+ female mice.

### Brain ^54^Mn levels after intravenous injection

**Fig. 4:**
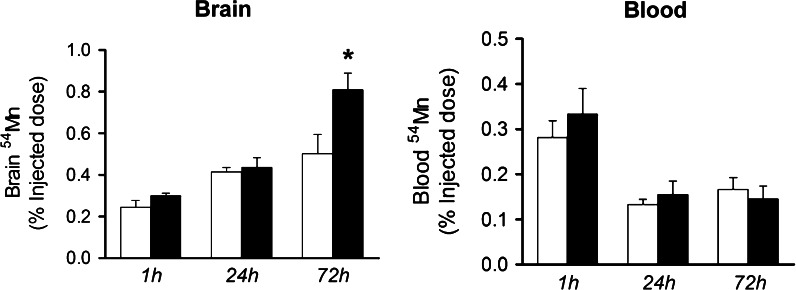
Effect of Fpn deficiency on Mn uptake after intravenous injection. Levels of ^54^Mn in brain and blood were characterized 1, 24, or 72 h post-dose of ^54^MnCl_2_ to mice by intravenous injection. *Empty and closed bars* represent +*/*+ and ffe/+ mice, respectively. Data were presented as mean ± SEM (n = 5/group; 3 male and 2 female). *P < 0.05 between +*/*+ versus *ffe/*+ mice; *t*-test

Amongst the metals studied, Mn is known to accumulate in the brain to produce neurobehavioral effects (Kern and Smith 2011; Kern et al. 2010). Therefore, to assess the distribution of this metal from the blood to the brain we studied the influence of Fpn deficiency on brain Mn accumulation after intravenous injection of ^54^MnCl_2_. ^54^Mn levels in the brain increased in a time-dependent manner for both *flatiron* and wild-type mice. Levels of ^54^Mn deposition was greater in *ffe/*+ at 72 h (Fig. 4). ^54^Mn clearance from the blood to peripheral tissues after intravenous metal absorption occurred rapidly such that similar levels of the radioisotope are found 24 and 72 h post-injection in both *ffe/*+ and +*/*+ mice. Except for the brain, significant differences in ^54^Mn deposition into other tissues were not detected (data not shown).

## Discussion

The present study was undertaken to investigate the influence of Fpn deficiency on the distribution of metals in the *flatiron* mouse model of “ferroportin disease”. This disorder is known as type IV hemochromatosis, and arises from mutations in the human Fpn (SLC40A1) gene. While clinical manifestations of the disease are quite variable, most mutations are inherited as dominant traits. Over 35 polymorphisms are known (Pietrangelo et al. 2011). Ferroportin disease is associated with mild anemia and Fe loading in human patients. Consistent with previous studies (Seo and Wessling-Resnick 2015; Zohn et al. 2007), *flatiron* mice used in this investigation displayed features of both Fe-restricted erythropoiesis and Fe loading in liver and spleen, recapitulating the human disease (Pietrangelo 2004).

Both heterozygous *ffe/*+ and +*/*+ mice weanlings were fed a diet containing adequate levels to meet but not exceed nutritional requirements for these essential yet toxic minerals. Adjusting for reduced haematocrit, analysis of metal contents in red blood cells and plasma did not indicate differences between *flatiron* and wild-type mice in Zn or Cu. However, the level of circulating Mn associated with the cell fraction was significantly reduced in *ffe/*+ mice compared to +*/*+ mice. This observation is consistent with our earlier study that showed reduced total blood Mn in heterozygous *flatiron* mice as well as reduced red blood cell superoxide dismutase activity (Seo and Wessling-Resnick 2015).

Mn is essential for bone health. Along with other minerals, it is incorporated into calcified tissue (Gdula-Argasinska et al. 2004). Mn-deficient diets produce impaired osteoclast activity, alter bone resorption and skeletal development (Aschner and Aschner 2005). It has been estimated that nearly 40 % of body Mn is found in bone, and that excess Mn increases bone content (O’Neal et al. 2014). *Flatiron* mice display reduced bone Mn content, consistent with the Mn deficiency demonstrated by reduced blood Mn and superoxide dismutase activity (Seo and Wessling-Resnick 2015). While Fe levels in bone did not change, Zn and Cu were both reduced in femurs from *ffe*/+ mice compared to +*/*+ mice. Moreover, Cu levels in muscle were reduced in *ffe*/+ mice compared to +*/*+ mice. Interestingly, kidney Zn and Cu were also reduced in *ffe*/+ mice compared to +*/*+ mice. These combined findings show that Fpn deficiency can impact the distribution of Zn and Cu between various organs in the body. They also point to a possible role of Fpn in resorption or re-absorption of these metals by bone and kidneys. Because bone is a good long-term biomarker for metal status, the reduced levels of Mn, Zn and Cu support the idea that in addition to Fe, other biometals become depleted in ferroportin disease. While limited in vitro evidence supports a role for Fpn in transport of other biometals, the flatiron mice provide the first in vivo model to test this hypothesis. Although the simplest hypothesis is that Fpn itself mediates export of these other metals, the possibility that loss of iron transport by Fpn affects other metal regulatory systems cannot be excluded.

Fpn is not only highly expressed in macrophages of the reticuloendothelial system, intestinal duodenum, and hepatocytes (Donovan et al. 2000, 2005; Knutson et al. 2005), but it is also ubiquitously expressed in murine brain (Boserup et al. 2011), including the olfactory region (Kim et al. 2013). Both Mn and Fe accumulated in the olfactory bulbs of *flatiron* mice. Although Zn levels were also higher, values were not statistically different (*P* = 0.06). Brain Mn metabolism is of particular concern since this metal is neurotoxic (Roels et al. 2012). A significant route of entry is uptake of airborne Mn since this metal is efficiently transported into the body through the nasal epithelium (Brenneman et al. 2000; Nong et al. 2008; Tjalve et al. 1996). The accumulation of Mn and Fe in the olfactory bulbs of *flatiron* mice suggests that deficiency of Fpn export function may exacerbate neurotoxicity caused by exposure to these metals.

To evaluate the role of Fpn in brain metal accumulation, we studied the distribution of ^54^Mn after intravenous injection and found that levels of radioisotope were significantly increased in the brain of *flatiron* mice 72 h post-injection while circulating levels were similar to control mice. These data suggest that Fpn deficiency in *flatiron* mice enhances accumulation of ^54^Mn in the brain and are consistent with the ICP-MS analysis indicating tissue metal levels in brains of the heterozygous offspring are generally higher and significantly greater in the olfactory bulb region. Results of our study indicate that Fpn plays a role in maintaining steady-state levels of Mn in the brain and suggest it mediates Mn export from the brain via the vascular pathway. Further studies are necessary to more fully define the role of Fpn in blood–brain transport of Mn and how it contributes to the observed increases in olfactory bulb and brain metal content.

A major finding from our study is that in addition to Mn and Fe, aspects of Zn and Cu transport and metabolism appear to be affected in the murine model of ferroportin disease. For example, reduced Zn and Cu levels in *flatiron* kidneys imply an important role for Fpn in renal function. On the other hand, reduced levels of Mn in the lung could help protect against infection since this metal has been shown to be critical to the cellular physiology of pneumococcus (Hood and Skaar 2012; Kehl-Fie and Skaar 2010) and studies have shown increased pulmonary susceptibility in mice to streptococcal infection after Mn inhalation (Adkins et al. 1980). More problematic would be the accumulation of Mn and other biometals in brain and the olfactory bulb discussed above. Patients with ferroportin disease may be more susceptible to metal neurotoxicity. Ferroportin disease arises due to several different missense mutations in the Fpn gene, and is the only type of hemochromatosis that has a dominant transmission pattern (Pietrangelo 2004). The disease has clinical heterogeneity, with marked differences in type of target cell and Fe accumulation, sub-phenotypes and degree of penetrance (Pietrangelo 2004). Ferroportin disease is the second most common cause of hereditary hemochromatosis (Pietrangelo 2004). Chronic Mn exposure results in metal accumulation in specific brain regions associated with Parkinsonian motor dysfunction (Guilarte 2010; Racette et al. 2001). Our study raises the possibility that patients with ferroportin disease are more susceptible to Mn neurotoxicity and neurodegenerative diseases associated with metal toxicity. In support of this concept, our recent studies using an in vitro expression system revealed that ferroportin provides neuroprotection against Mn toxicity, while flatiron mutant H32R failed to confer protection (Seo and Wessling-Resnick 2015). These studies suggest that loss of function mutant H32R leads to increased susceptibility to Mn toxicity at the cellular level. We are currently exploring the influence of loss of function mutants in ferroportin disease on metal toxicity in mice.

## Electronic supplementary material

Supplementary material 1 (DOCX 182 kb)
