# Supplementary material for: Distribution of manganese and other biometals in flatiron mice
Source: Biometals. 2015 Dec 22;29:147–55. doi: 10.1007/s10534-015-9904-2 (PMC4735247; doi:10.1007/s10534-015-9904-2)
Supplement: Supplementary file 1 — Supplementary material 1 (DOCX 182 kb) [file 10534_2015_9904_MOESM1_ESM.docx]

**Supplemental Table 1** Tissue metal levels in mice

Data from previous studies of mice are summarized. The tilde symbol (~) indicates approximate values from the graph reported in previous studies.

| **Organ** | **Metal** | **Metal content  (ug/g)** | **Mouse Model** | **Age  (weeks)** | **Gender** | **Reference** |
| --- | --- | --- | --- | --- | --- | --- |
| **Serum** | **Mn** | 0.0059 | Balb/c | 8-10 | Female | ([1](#_ENREF_1)) |
|  |  | 0.0054 | Balb/c | 6 | Female | ([1](#_ENREF_1)) |
|  |  | 0.0142 | C57BL/6 | N/A | Female | ([2](#_ENREF_2)) |
|  | **Fe** | ~3 | 129S6/SvEvTac | 5 | Male and Female | ([3](#_ENREF_3)) |
|  |  | 5.817 | Balb/c | 6 | Female | ([1](#_ENREF_1)) |
|  |  | 4.1 | Balb/c | 8-10 | Female | ([1](#_ENREF_1)) |
|  |  | 14.8 | C57BL/6 | N/A | Female | ([2](#_ENREF_2)) |
|  | **Cu** | 0.441 | Balb/c | 6 | Female | ([1](#_ENREF_1)) |
|  |  | 0.462 | Balb/c | 8-10 | Female | ([1](#_ENREF_1)) |
|  |  | 0.632 | C57BL/6 | N/A | Female | ([2](#_ENREF_2)) |
|  | **Zn** | 1.183 | Balb/c | 6 | Female | ([1](#_ENREF_1)) |
|  |  | 0.8 | Balb/c | 8-10 | Female | ([1](#_ENREF_1)) |
|  |  | 0.983 | C57BL/6 | N/A | Female | ([2](#_ENREF_2)) |
| **Plasma** | **Fe** | ~4.5 | Hsd:ICR (CD1) | 4 | Male | ([4](#_ENREF_4)) |
|  | **Cu** | ~1.15 | Balb/c | N/A | Female | ([5](#_ENREF_5)) |
|  |  | ~1.12 | C57BL/6 | N/A | Female | ([5](#_ENREF_5)) |
|  | **Zn** | ~1.12 | Balb/c | N/A | Female | ([5](#_ENREF_5)) |
|  |  | ~1.1 | C57BL/6 | N/A | Female | ([5](#_ENREF_5)) |
| **Bone** | **Mn** | ~0.9 | C57BL/6J | 16 | N/A | ([6](#_ENREF_6)) |
|  | **Fe** | ~50 | C57BL/6J | 16 | N/A | ([6](#_ENREF_6)) |
|  | **Cu** | ~1 | C57BL/6J | 16 | N/A | ([6](#_ENREF_6)) |
|  | **Zn** | 212 | C57BL/Ks | 8 - 12 | N/A | ([7](#_ENREF_7)) |
|  |  | ~50 | C57BL/6J | 16 | N/A | ([6](#_ENREF_6)) |
|  |  | 183 | C57BL/KsJ | 17-18 | Female | ([8](#_ENREF_8)) |
| **Muscle** | **Mn** | ~0.2 | C57BL/6 | 12 | Male | ([9](#_ENREF_9)) |
|  |  | ~0.12 | C57BL/6J | 16 | N/A | ([6](#_ENREF_6)) |
|  | **Fe** | ~20 | C57BL/6J | 16 | N/A | ([6](#_ENREF_6)) |
|  | **Cu** | ~0.75 | C57BL/6 | 12 | Male | ([9](#_ENREF_9)) |
|  |  | ~1 | C57BL/6J | 16 | N/A | ([6](#_ENREF_6)) |
|  | **Zn** | 14.8 | Balb/c | 8 - 12 | Male | ([10](#_ENREF_10)) |
|  |  | ~8 | C57BL/6 | 12 | Male | ([9](#_ENREF_9)) |
|  |  | ~9 | C57BL/6J | 16 | N/A | ([6](#_ENREF_6)) |
| **Liver** | **Mn** | ~0.9 | C57BL/6 | 4 - 28 | Male and Female | ([11](#_ENREF_11)) |
|  |  | 1.08 | Swiss albino | 6 - 7 | Male | ([12](#_ENREF_12)) |
|  |  | ~1.8 | Kunming | 11.5-13.5 | N/A | ([13](#_ENREF_13)) |
|  |  | ~1 | C57BL/6 | 12 | Male | ([9](#_ENREF_9)) |
|  |  | 0.518 | C57BL/6J | N/A | Female | ([2](#_ENREF_2)) |
|  |  | ~1.2 | C57BL/6J X C3/HeJ | N/A | N/A | ([14](#_ENREF_14)) |
|  | **Fe** | 111 | Hsd:ICR (CD1) | 4 | Male | ([4](#_ENREF_4)) |
|  |  | 101 | Crl:CFW(SW) | 4 | Male | ([15](#_ENREF_15)) |
|  |  | ~100 | C57BL/6 | 4 - 28 | Male and Female | ([11](#_ENREF_11)) |
|  |  | ~280 | ICR (CD-1) | 6 | Female | ([16](#_ENREF_16)) |
|  |  | 55.62 | Swiss albino | 6 - 7 | Male | ([12](#_ENREF_12)) |
|  |  | ~110 | CBA/C3H | 11 | N/A | ([17](#_ENREF_17)) |
|  |  | ~100 | C57BL/6J | 16 | N/A | ([6](#_ENREF_6)) |
|  |  | 136 | C57BL/6J | N/A | Female | ([2](#_ENREF_2)) |
|  |  | ~100 | C57BL/6J X C3/HeJ | N/A | N/A | ([14](#_ENREF_14)) |
|  |  | ~80 | Kunming | N/A | Male and Female | ([18](#_ENREF_18)) |
|  | **Cu** | 4.27 | Hsd:ICR (CD1) | 4 | Male | ([4](#_ENREF_4)) |
|  |  | 2.01 | Crl:CFW(SW) | 4 | Male | ([15](#_ENREF_15)) |
|  |  | 3.63 | Swiss albino | 6 - 7 | Male | ([12](#_ENREF_12)) |
|  |  | ~3 | CBA/C3H | 11 | N/A | ([17](#_ENREF_17)) |
|  |  | ~9.8 | Kunming | 11.5-13.5 | N/A | ([13](#_ENREF_13)) |
|  |  | ~5 | C57BL/6 | 12 | Male | ([9](#_ENREF_9)) |
|  |  | ~5.8 | C57BL/6J | 16 | N/A | ([6](#_ENREF_6)) |
|  |  | 4 | C57BL/6J | N/A | Female | ([2](#_ENREF_2)) |
|  |  | ~3 | CRL:CD1 (ICR) | N/A | Female | ([19](#_ENREF_19)) |
|  |  | ~9 | C57BL/6J X C3/HeJ | N/A | N/A | ([14](#_ENREF_14)) |
|  |  | ~4.4 | Kunming | N/A | Male and Female | ([18](#_ENREF_18)) |
|  | **Zn** | 22.47 | Swiss albino | 6 - 7 | Male | ([12](#_ENREF_12)) |
|  |  | 42.6 | Balb/c | 8 - 12 | Male | ([10](#_ENREF_10)) |
|  |  | 99.5 | C57BL/Ks | 8 - 12 | N/A | ([7](#_ENREF_7)) |
|  |  | ~62 | Kunming | 11.5-13.5 | N/A | ([13](#_ENREF_13)) |
|  |  | ~24 | C57BL/6 | 12 | Male | ([9](#_ENREF_9)) |
|  |  | ~27 | C57BL/6J | 16 | N/A | ([6](#_ENREF_6)) |
|  |  | 25.8 | C57BL/KsJ | 17-18 | Female | ([8](#_ENREF_8)) |
|  |  | 30.8 | C57BL/6J | N/A | Female | ([2](#_ENREF_2)) |
|  |  | ~30 | C57BL/6J X C3/HeJ | N/A | N/A | ([14](#_ENREF_14)) |
|  |  | ~25 | Kunming | N/A | Male and Female | ([18](#_ENREF_18)) |
| **Spleen** | **Mn** | ~0.12 | C57BL/6J | 16 | N/A | ([6](#_ENREF_6)) |
|  | **Fe** | ~1100 | C57BL/6 | 4 - 28 | Male and Female | ([11](#_ENREF_11)) |
|  |  | ~840 | ICR (CD-1) | 6 | Female | ([16](#_ENREF_16)) |
|  |  | ~800 | C57BL/6J | 16 | N/A | ([6](#_ENREF_6)) |
|  | **Cu** | ~1.1 | C57BL/6J | 16 | N/A | ([6](#_ENREF_6)) |
|  |  | ~1 | CRL:CD1 (ICR) | N/A | Female | ([19](#_ENREF_19)) |
|  | **Zn** | 27.4 | Balb/c | 8 - 12 | Male | ([10](#_ENREF_10)) |
|  |  | ~19 | C57BL/6J | 16 | N/A | ([6](#_ENREF_6)) |
| **Kidney** | **Mn** | 1.04 | Swiss albino | 6 - 7 | Male | ([12](#_ENREF_12)) |
|  | **Fe** | ~50 | C57BL/6 | 4 - 28 | Male and Female | ([11](#_ENREF_11)) |
|  |  | ~130 | ICR (CD-1) | 6 | Female | ([16](#_ENREF_16)) |
|  |  | 68.73 | Swiss albino | 6 - 7 | Male | ([12](#_ENREF_12)) |
|  |  | ~50 | C57BL/6J | 16 | N/A | ([6](#_ENREF_6)) |
|  |  | ~90 | Kunming | N/A | Male and Female | ([18](#_ENREF_18)) |
|  | **Cu** | 3.61 | Swiss albino | 6 - 7 | Male | ([12](#_ENREF_12)) |
|  |  | ~2.7 | C57BL/6J | 16 | N/A | ([6](#_ENREF_6)) |
|  |  | ~3.1 | CRL:CD1 (ICR) | N/A | Female | ([19](#_ENREF_19)) |
|  |  | ~4.6 | Kunming | N/A | Male and Female | ([18](#_ENREF_18)) |
|  | **Zn** | 15.45 | Swiss albino | 6 - 7 | Male | ([12](#_ENREF_12)) |
|  |  | ~23.3 | Balb/c | 8 - 12 | Male | ([10](#_ENREF_10)) |
|  |  | 86.4 | C57BL/Ks | 8 - 12 | N/A | ([7](#_ENREF_7)) |
|  |  | ~13 | C57BL/6J | 16 | N/A | ([6](#_ENREF_6)) |
|  |  | ~30 | Kunming | N/A | Male and Female | ([18](#_ENREF_18)) |
| **Lung** | **Mn** | 0.25 | Swiss albino | 6 - 7 | Male | ([12](#_ENREF_12)) |
|  |  | ~0.1 | C57BL/6J | 16 | N/A | ([6](#_ENREF_6)) |
|  | **Fe** | 85.94 | Swiss albino | 6 - 7 | Male | ([12](#_ENREF_12)) |
|  |  | ~200 | C57BL/6J | 16 | N/A | ([6](#_ENREF_6)) |
|  | **Cu** | 1.81 | Swiss albino | 6 - 7 | Male | ([12](#_ENREF_12)) |
|  |  | ~1.8 | C57BL/6J | 16 | N/A | ([6](#_ENREF_6)) |
|  |  | ~2.3 | CRL:CD1 (ICR) | N/A | Female | ([19](#_ENREF_19)) |
|  | **Zn** | ~22 | C57BL/6J | 16 | N/A | ([6](#_ENREF_6)) |
|  |  | 20.3 | Swiss albino | 6 - 7 | Male | ([12](#_ENREF_12)) |
| **Heart** | **Mn** | 0.55 | Swiss albino | 6 - 7 | Male | ([12](#_ENREF_12)) |
|  |  | 0.4 | C57BL/6J | 16 | N/A | ([6](#_ENREF_6)) |
|  |  | ~0.309 | C57BL/6J | N/A | Female | ([2](#_ENREF_2)) |
|  | **Fe** | 245.61 | Swiss albino | 6 - 7 | Male | ([12](#_ENREF_12)) |
|  |  | ~100 | C57BL/6 | 4 - 28 | Male and Female | ([11](#_ENREF_11)) |
|  |  | ~100 | C57BL/6J | 16 | N/A | ([6](#_ENREF_6)) |
|  |  | 87.5 | C57BL/6J | N/A | Female | ([2](#_ENREF_2)) |
|  | **Cu** | 3.57 | Swiss albino | 6 - 7 | Male | ([12](#_ENREF_12)) |
|  |  | ~5.2 | C57BL/6J | 16 | N/A | ([6](#_ENREF_6)) |
|  |  | 6.5 | C57BL/6J | N/A | Female | ([2](#_ENREF_2)) |
|  | **Zn** | 13.64 | Swiss albino | 6 - 7 | Male | ([12](#_ENREF_12)) |
|  |  | 20.5 | Balb/c | 8 - 12 | Male | ([10](#_ENREF_10)) |
|  |  | ~18 | C57BL/6J | 16 | N/A | ([6](#_ENREF_6)) |
|  |  | 18 | C57BL/6J | N/A | Female | ([2](#_ENREF_2)) |
| **Pancreas** | **Mn** | 1.656 | Balb/c | 6 | Female | ([1](#_ENREF_1)) |
|  |  | ~1.3 | C57BL/6J | 16 | N/A | ([6](#_ENREF_6)) |
|  | **Fe** | 45.299 | Balb/c | 6 | Female | ([1](#_ENREF_1)) |
|  |  |  |  |  |  |  |
|  | **Cu** | 1.342 | Balb/c | 6 | Female | ([1](#_ENREF_1)) |
|  |  | ~1.9 | C57BL/6J | 16 | N/A | ([6](#_ENREF_6)) |
|  |  |  |  |  |  |  |
|  | **Zn** | 35.218 | Balb/c | 6 | Female | ([1](#_ENREF_1)) |
|  |  | ~34 | C57BL/6J | 16 | N/A | ([6](#_ENREF_6)) |
| **Olfactory Bulb** | **Mn** | ~0.5  (8 bulbs pooled) | 129S6/SvEvTac | 5 | Male and Female | ([3](#_ENREF_3)) |
|  |  | ~27  (8 bulbs pooled) | 129S6/SvEvTac | 5 | Male and Female | ([3](#_ENREF_3)) |
|  | **Cu** | ~4.2 | CRL:CD1 (ICR) | N/A | female | ([19](#_ENREF_19)) |
| **Brain** | **Mn** | 0.39 | Swiss albino | 6 - 7 | Male | ([12](#_ENREF_12)) |
|  |  | 0.315 | Balb/c | 8 - 10 | Female | ([1](#_ENREF_1)) |
|  |  | ~0.4 | C57BL/6 | 12 | Male | ([9](#_ENREF_9)) |
|  |  | ~0.46 | C57BL6/SJL | 15.7 | Male and Female | ([20](#_ENREF_20)) |
|  |  | ~0.4 | C57BL/6J X C3/HeJ | N/A | N/A | ([14](#_ENREF_14)) |
|  | **Fe** | ~11.5 | Hsd:ICR (CD1) | 4 | Male | ([4](#_ENREF_4)) |
|  |  | ~20 | C57BL/6J | 4 - 28 | Male and Female | ([11](#_ENREF_11)) |
|  |  | 17.62 | Swiss albino | 6 - 7 | Male | ([12](#_ENREF_12)) |
|  |  | 22.4 | Balb/c | 8 - 10 | Female | ([1](#_ENREF_1)) |
|  |  | ~12 | C57BL/6 | 12 | Male | ([9](#_ENREF_9)) |
|  |  | ~17 | C57BL6/SJL | 15.7 | Male and Female | ([20](#_ENREF_20)) |
|  |  | ~20 | C57BL/6J X C3/HeJ | N/A | N/A | ([14](#_ENREF_14)) |
|  | **Cu** | 3.12 | Hsd:ICR (CD1) | 4 | Male | ([4](#_ENREF_4)) |
|  |  | ~3.9 | C57BL/6J | 4 - 28 | Male and Female | ([11](#_ENREF_11)) |
|  |  | 2.52 | Swiss albino | 6 - 7 | Male | ([12](#_ENREF_12)) |
|  |  | 4.3 | Balb/c | 8 - 10 | Female | ([1](#_ENREF_1)) |
|  |  | ~3.6 | C57BL/6 | 12 | Male | ([9](#_ENREF_9)) |
|  |  | ~4.5 | C57BL6/SJL | 15.7 | Male and Female | ([20](#_ENREF_20)) |
|  |  | 5.45 | C57BL/6J x 129 | 15-24 | Male | ([21](#_ENREF_21)) |
|  | **Cu** | ~8 | B6SJL-Tg(SOD1-G93A)1Gur/J | N/A | N/A | ([14](#_ENREF_14)) |
|  | **Zn** | 13.25 | Swiss albino | 6 - 7 | Male | ([12](#_ENREF_12)) |
|  |  | 17 | Balb/c | 8 - 10 | Female | ([1](#_ENREF_1)) |
|  |  | 15.4 | Balb/c | 8 - 12 | Male | ([10](#_ENREF_10)) |
|  |  | ~13 | C57BL/6 | 12 | Male | ([9](#_ENREF_9)) |
|  |  | ~20 | C57BL6/SJL | 15.7 | Male and Female | ([20](#_ENREF_20)) |
|  |  | ~22 | C57BL/6J X C3/HeJ | N/A | Male and Female | ([14](#_ENREF_14)) |

1. Ilback NG, Benyamin G, Lindh U, Fohlman J, Friman G. Trace element changes in the pancreas during viral infection in mice. Pancreas 2003;26:190-6.

2. Edvinsson M, Frisk P, Molin Y, Hjelm E, Ilback NG. Trace element balance is changed in infected organs during acute Chlamydophila pneumoniae infection in mice. Biometals 2008;21:229-37.

3. Kim J, Buckett PD, Wessling-Resnick M. Absorption of manganese and iron in a mouse model of hemochromatosis. PLoS One 2013;8:e64944.

4. Pyatskowit JW, Prohaska JR. Rodent brain and heart catecholamine levels are altered by different models of copper deficiency. Comp Biochem Physiol C Toxicol Pharmacol 2007;145:275-81.

5. Amini M, Nahrevanian H, Khatami S, Farahmand M, Mirkhani F, Javadian S. Biochemical association between essential trace elements and susceptibility to Leishmania major in BALB/c and C57BL/6 mice. Braz J Infect Dis 2009;13:83-5.

6. Herrera C, Pettiglio MA, Bartnikas TB. Investigating the role of transferrin in the distribution of iron, manganese, copper, and zinc. J Biol Inorg Chem 2014;19:869-77.

7. Levine AS, McClain CJ, Handwerger BS, Brown DM, Morley JE. Tissue zinc status of genetically diabetic and streptozotocin-induced diabetic mice. Am J Clin Nutr 1983;37:382-6.

8. Donaldson DL, Kubo C, Smith CC, Good RA. Effects of genetic diabetes and zinc nutriture on in vivo cell-mediated immunity in the mouse. Am J Clin Nutr 1986;43:263-71.

9. Dang TN, Lim NK, Grubman A, Li QX, Volitakis I, White AR, Crouch PJ. Increased metal content in the TDP-43(A315T) transgenic mouse model of frontotemporal lobar degeneration and amyotrophic lateral sclerosis. Front Aging Neurosci 2014;6:15.

10. Verbanac D, Milin C, Domitrovic R, Giacometti J, Pantovic R, Ciganj Z. Determination of standard zinc values in the intact tissues of mice by ICP spectrometry. Biol Trace Elem Res 1997;57:91-6.

11. Lu S, Seravalli J, Harrison-Findik D. Inductively coupled mass spectrometry analysis of biometals in conditional Hamp1 and Hamp1 and Hamp2 transgenic mouse models. Transgenic Res 2015.

12. Doker S, Mounicou S, Dogan M, Lobinski R. Probing the metal-homeostatis effects of the administration of chromium(vi) to mice by ICP MS and size-exclusion chromatography-ICP MS. Metallomics 2010;2:549-55.

13. Wang X, Wang H, Li J, Yang Z, Zhang J, Qin Z, Wang L, Kong X. Evaluation of bioaccumulation and toxic effects of copper on hepatocellular structure in mice. Biol Trace Elem Res 2014;159:312-9.

14. Lelie HL, Liba A, Bourassa MW, Chattopadhyay M, Chan PK, Gralla EB, Miller LM, Borchelt DR, Valentine JS, Whitelegge JP. Copper and zinc metallation status of copper-zinc superoxide dismutase from amyotrophic lateral sclerosis transgenic mice. J Biol Chem 2011;286:2795-806.

15. Prohaska JR, Brokate B. The timing of perinatal copper deficiency in mice influences offspring survival. J Nutr 2002;132:3142-5.

16. Antonelli A, Sfara C, Battistelli S, Canonico B, Arcangeletti M, Manuali E, Salamida S, Papa S, Magnani M. New strategies to prolong the in vivo life span of iron-based contrast agents for MRI. PLoS One 2013;8:e78542.

17. Gulec S, Collins JF. Investigation of iron metabolism in mice expressing a mutant Menke's copper transporting ATPase (Atp7a) protein with diminished activity (Brindled; Mo (Br) (/y) ). PLoS One 2013;8:e66010.

18. Suhua W, Rongzhu L, Changqing Y, Guangwei X, Fangan H, Junjie J, Wenrong X, Aschner M. Lipid peroxidation and changes of trace elements in mice treated with paradichlorobenzene. Biol Trace Elem Res 2010;136:320-36.

19. Zhang L, Bai R, Liu Y, Meng L, Li B, Wang L, Xu L, Le Guyader L, Chen C. The dose-dependent toxicological effects and potential perturbation on the neurotransmitter secretion in brain following intranasal instillation of copper nanoparticles. Nanotoxicology 2012;6:562-75.

20. Maynard CJ, Cappai R, Volitakis I, Cherny RA, White AR, Beyreuther K, Masters CL, Bush AI, Li QX. Overexpression of Alzheimer's disease amyloid-beta opposes the age-dependent elevations of brain copper and iron. J Biol Chem 2002;277:44670-6.

21. Waggoner DJ, Drisaldi B, Bartnikas TB, Casareno RL, Prohaska JR, Gitlin JD, Harris DA. Brain copper content and cuproenzyme activity do not vary with prion protein expression level. J Biol Chem 2000;275:7455-8.
